# Sexual, allometric and forest cover effects on giant anteaters’ movement ecology

**DOI:** 10.1371/journal.pone.0253345

**Published:** 2021-08-18

**Authors:** Aline Giroux, Zaida Ortega, Luiz Gustavo Rodrigues Oliveira-Santos, Nina Attias, Alessandra Bertassoni, Arnaud Léonard Jean Desbiez

**Affiliations:** 1 Ecology Department, Federal University of Mato Grosso do Sul (UFMS), Campo Grande, Mato Grosso do Sul, Brazil; 2 Zoology Department, University of Granada, Granada, Granada, Spain; 3 Institute for the Conservation of Wild Animals (ICAS), Campo Grande, Mato Grosso do Sul, Brazil; 4 Ecology and Evolution Department, Federal University of Goiás (UFG), Goiânia, Goiás, Brazil; 5 Institute for Research and Conservation of Tamanduas in Brazil (Tamanduá Institute), Parnaíba, Piauí, Brazil; 6 Institute for Ecological Research (IPÊ), Nazaré Paulista, São Paulo, Brazil; 7 Royal Zoological Society of Scotland (RZSS), Murrayfield, Edinburgh, United Kingdom; Sichuan University, CHINA

## Abstract

Knowing the influence of intrinsic and environmental traits on animals’ movement is a central interest of ecology and can aid to enhance management decisions. The giant anteater (*Myrmecophaga tridactyla*) is a vulnerable mammal that presents low capacity for physiological thermoregulation and uses forests as thermal shelters. Here, we aim to provide reliable estimates of giant anteaters’ movement patterns and home range size, as well as untangle the role of intrinsic and environmental drivers on their movement. We GPS-tracked 19 giant anteaters in Brazilian savannah. We used a continuous-time movement model to estimate their movement patterns (described by home range crossing time, daily distance moved and directionality), and provide an autocorrelated kernel density estimate of home range size. Then, we used mixed structural equations to integratively model the effects of sex, body mass and proportion of forest cover on movement patterns and home range size, considering the complex net of interactions between these variables. Male giant anteaters presented more intensive space use and larger home range than females with similar body mass, as it is expected in polygynous social mating systems. Males and females increased home range size with increasing body mass, but the allometric scaling of intensity of space use was negative for males and positive for females, indicating different strategies in search for resources. With decreasing proportion of forest cover inside their home ranges, and, consequently, decreasing thermal quality of their habitat, giant anteaters increased home range size, possibly to maximize the chances of accessing thermal shelters. As frequency and intensity of extreme weather events and deforestation are increasing, effective management efforts need to consider the role of forests as an important thermal resource driving spatial requirements of this species. We highlight that both intrinsic and environmental drivers of animal movement should be integrated to better guide management strategies.

## Introduction

Animal movement is a key process of ecology, driving animals’ survival and fitness [1]. The individuals’ movement patterns shape their home range, which can be defined as the part of their cognitive map that they choose to be continuously updated [2, 3]. The home range should offer the needed conditions for basic activities of food gathering, mating, and caring for young [4]. Describing movement patterns and quantifying the home range size allow us to better understand the ecology and spatial requirements of animals to make appropriate management decisions that can help to preserve wildlife populations [5, 6]. Such knowledge has become even more important as human actions are increasingly endangering natural systems [7, 8]. Theoretical and empirical studies have more often focused on understanding ‘typical’ movement of a species than understanding its variation [9]. However, both movement patterns and home range size widely vary between individuals within a population, and these variations are commonly influenced by intrinsic and environmental traits [5, 7].

Sex and body mass are among the main intrinsic traits driving intraspecific variations on animal movement [10]. The mating system and the associated reproductive tactics employed by males and females within a species influence the evolutionary selection of various characters [11]. This can result in sex-related metabolic, cognitive, and behavioral differences that should be reflected on movement patterns and, consequently, on home range size [12]. Body mass, in turn, has direct influence on the individual’s body mechanics and physiology [13, 14]. Besides, body mass can drive movement patterns and home range size by influencing, for example, the energetic requirements of individuals [15, 16], the foraging experience [17], and/or the orientation ability [18]. Therefore, the animal movement allometry relative to individuals’ body mass is commonly found [13–18]. Among environmental traits shaping animal movement patterns and home range size, the proportion of available forest cover stands out to animals that habit fragmented landscapes and use forests as a resource [19–21].

The influence of intrinsic and environmental traits on animal movement is being increasingly better understood as technological advances on tracking methods increase [9]. Although the analysis of movement data is still challenging [22, 23], the recent implementation of continuous-time movement models on understanding movement patterns and on estimating home range size has allowed great advances [24, 25]. For high-quality GPS tracking data of range-resident individuals, these models allow the estimation of descriptors of movement patterns such as home range crossing-time, daily distance moved and directionality [26]. These descriptors bring insights on underlying movement processes determining home range and can be used to provide an autocorrelated kernel density estimator of home range size [26]. However, previous research has focused on evaluating one specific movement metric at a time [27, 28], disregarding the possible causal relationships of the descriptors of movement patterns with each other and with home range size. Both accurate estimates of animal movement, based on movement models, and integrative approaches that consider the complex network of relations between the variables can help us to understand the effect of intrinsic and environmental traits on movement patterns and home range size.

The giant anteater (*Myrmecophaga tridactyla*) is a vulnerable mammal whose movement patterns and home range size have been previously studied to better guide its management and conservation [29, 30]. Their original spatial distribution covered from Belize to the south of South America, excluding the Andes [29]. While some populations are already locally extinct, others are facing habitat loss, wildfires, roadkills, conflicts with dogs and other threats [29]. In this scenario, their low reproductive rate and long periods of parental care make giant anteaters conservation status even more worrisome [31, 32]. Despite being commonly associated with open habitats [33], forests have a fundamental role in giant anteater thermoregulation [34–36]. This is because giant anteaters present reduced body heat production [37] and low capacity of physiological thermoregulation, and forests act as important thermal shelters. Besides showing smaller environmental temperature variation than adjacent open areas, forest patches buffer rain and chilly winds and offer protection against solar radiation [38]. Therefore, it is also worrying that deforestation may be reducing the habitat thermal quality for these animals across their current distribution [39].

Despite the efforts to understand giant anteaters’ movement ecology, previous estimates of their home range size have ignored the intrinsic autocorrelation of high-resolution movement data and have not been based on movement models, probably generating underestimated results [24, 30]. While some studies showed no evidence of sexual effects on their movement [36, 40], other ones showed males presenting longer daily activity time [41, 42] and using larger areas than females [42]. Because of their sexual size dimorphism [36], the possible influence of body mass on movement needs to be considered when assessing sexual effects. Besides, although we know that giant anteaters select forests to set their home ranges and allocate time within it [42], we still ignore if the proportion of forest cover within home ranges influences their movement patterns and spatial requirements. Here we used a continuous-time movement model to offer reliable estimates of giant anteaters’ movement patterns (specifically home range crossing-time, daily distance moved and directionality) and home range size. Then, we investigated the effect of sex, body mass and proportion of forest cover on giant anteaters’ movement patterns and home range size. Using an integrative approach, we were able to uncover all these effects simultaneously, controlling for the possible relations among descriptors of movement pattern and with home range size.

**Fig 1:**
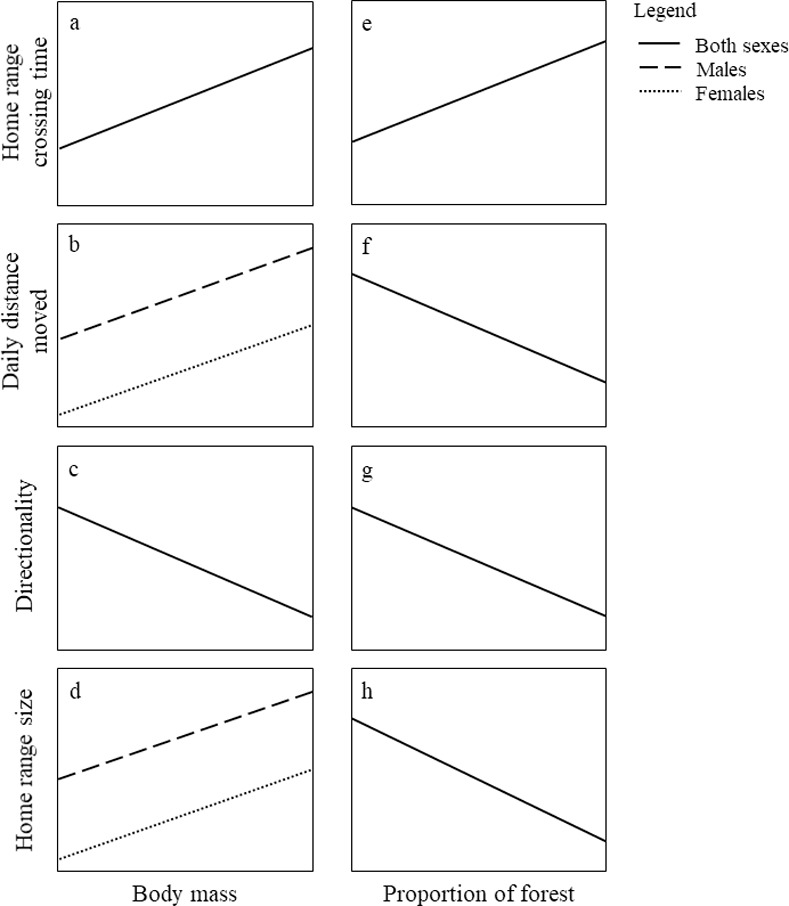
Hypothetical direction and shape of the expected effects of intrinsic (sex and body mass) and environmental traits (proportion of forest cover inside home range) on three descriptors of movement patterns (home range crossing time, daily distance moved and directionality) and home range size of giant anteaters (*Myrmecophaga tridactyla*).

Due to their probably polygynous social mating system [43], we expected male giant anteaters to increase their chances of mating opportunities by moving longer daily distances and using larger home ranges than females (Fig 1B and 1D) [44]. We also expected an allometric scaling between body mass and movement, since larger bodied individuals have higher energetic requirements than smaller ones [45]. Larger giant anteaters should increase the intensity of space use, increasing home range crossing-time and daily distance moved while decreasing directionality. This is because this increase in the intensity of space use should increase the individuals’ chances to find food resources–mainly ants and termites–spread on the landscape (Fig 1A–1C) [26, 45, 46]. Besides, it is reasonable to expect that larger animals will require more space to meet their energetic requirements [45], so they would also increase home range size with increasing body mass (Fig 1D). We expect that increasing the proportion of forest cover inside the home range will lead the animals to increase home range crossing time, decreasing daily distance moved and directionality, because the forests’ three-dimensional structure should present physical obstacles to displacement, imposing more friction than open grasslands (Fig 1E and 1G) [47]. Finally, lower proportions of forest inside home range would decrease the animal’s access to thermal shelters, decreasing the habitat thermal quality. This could lead animals to increase their spatial requirements, and, consequently, increase home range size (Fig 1H) [48].

## Methods

### Study site

We carried the study out in two savannah areas in the Brazilian territory: (1) Santa Barbara Ecological Station, São Paulo state (22° 48’ 59’’ S, 49° 14’ 12’’ W) and (2) Baía das Pedras Ranch, Mato Grosso do Sul state (19° 18’ 9" S, 55° 47’ 4" W). The study areas have a tropical climate, with rainy summers and dry winters [49]. The landscape of both studied areas is composed of mosaics of open grasslands, scrublands, savannahs, and woodlands [50, 51]. The landscape of Santa Barbara Ecological Station also includes anthropic elements, such as exotic forests of *Pinus* sp. and *Eucalyptus* sp., as well as highways [51]. Baía das Pedras Ranch is located within the Pantanal wetland, and it presents a naturally fragmented landscape with permanent and temporary salty and freshwater ponds, where open grasslands are subjected to seasonal flooding [50].

We classified the landscapes using georeferenced maps (LANDSAT 7 TM) and the MapBiomas database (Collection 5) [52]. To test the effect of the forest cover in movement patterns and home range size of giant anteaters, we summarized the observed habitats in two categories: forest or non-forest. Forest areas included woodland savannahs, woodlands, riparian forests, regenerating arboreal vegetation and exotic forests. Non-forest areas included open grasslands, scrublands, open savannahs, and areas without vegetation cover. We calculated the proportion of forest cover within each individual home range dividing the number of pixels classified as forest by the total number of pixels. We performed satellite image processing and supervised classifications using raster [53], maptools [54], and rgdal [55] packages available in the R environment [56].

### Capture and data collection

We searched for giant anteaters by horse or by pickup vehicle at low speed (maximum of 20 km/h). Once we saw the anteaters, we captured them using dip nets, dart-guns, or a blowpipe. Anteaters were immobilized and sedated following the protocol described by [42] in Santa Barbara Ecological Station and following the protocol described by [57] in Baía das Pedras Ranch. Each captured individual was sexed, weighted, and equipped with a global positioning system (GPS) harness during anesthesia. We conducted a T test [58] to compare the mean body mass between individuals of our two study areas. None of the tracking devices exceeded 3% of the animals’ body mass. All procedures were conducted in accordance with the Guidelines of the American Society of Mammalogists for the use of wild mammals in research [59] and were performed under the license numbers SISBIO 16010–1 and SISBIO 38326–5 (Chico Mendes Institute for Biodiversity Conservation). After completing their recovery from the anesthesia, we released the giant anteaters at the site of capture for movement GPS-tracking.

### Movement patterns and home range analysis

We described animals’ movement patterns and estimated home range size using the ctmm R package [24, 56, 60, 61]. We first examined the empirical variogram of each individual tracking data to check for an asymptote [26], as it is an evidence of range residence and a premise for the movement parameters estimation [61]. Because tracking data with such short sampling intervals are inherently autocorrelated, we also used the variogram to investigate the autocorrelation structure of data, obtaining starting values for the variance and autocorrelation timescales. Then, we fitted continuous-time movement models to the individuals’ location data via maximum likelihood. Among the fitted models, we included the Brownian motion model (BM), the Ornstein-Uhlenbeck model (OU), the Integrated OU model (IOU) and the Ornstein-Uhlenbeck-F model (OUF) [26]. We ranked the movement models based on the second order Akaike Information Criterion (AIC_C_) [62] and selected the one with the best fit for each individual anteater data set.

For those animals that better fitted OUF model, we obtained the three descriptors of individuals’ movement patterns: home range crossing time (timescale of autocorrelation in position), daily distance moved and directionality (direction persistence timescale), as well as their confidence intervals [24]. For those animals that showed range residence (i.e., better fitted OU or OUF models), we used the ninety-five per cent area corrected autocorrelated kernel density estimator (AKDEc 95%) to estimate the individuals’ home range size and its confidence limits. AKDEc is a nonparametric home-range estimator that assumes the data represent a sample from a nonstationary, autocorrelated, continuous movement process [63]. This estimator allows movement models to be fitted to data with different temporal structures (e.g., irregular sampling regime, gaps, and short sampling time). Also, AKDEc allows to compare home ranges of individuals with different monitoring times. This is because AKDEc extrapolates the data, basing itself on parameters of the model selected for each individual data set, to provide reliable home range estimates [63].

### Structural Equation Modeling

**Fig 2:**
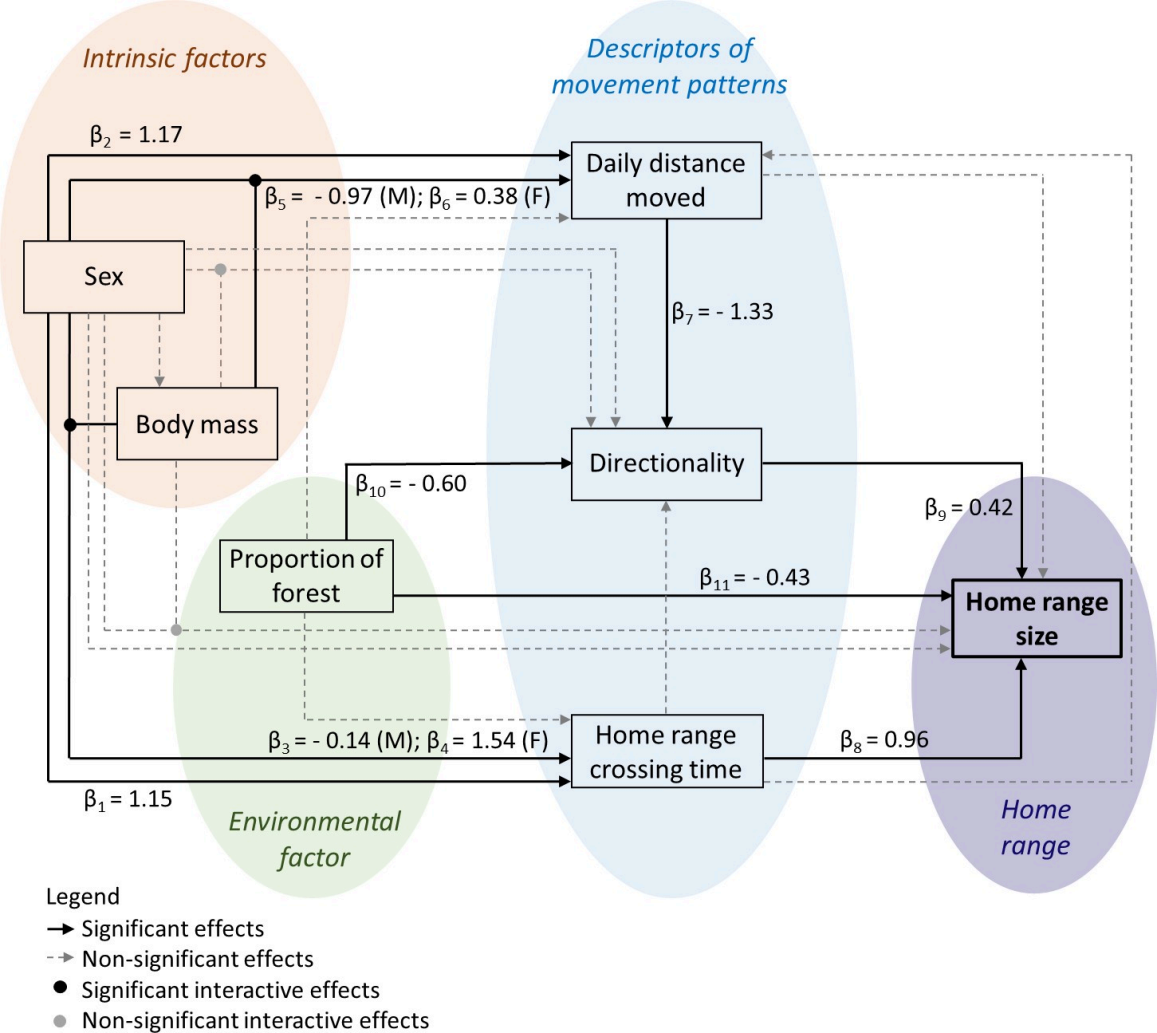
Schematic representation and estimated coefficients of the intrinsic (sex and body mass) and environmental effects (proportion of forest patches inside the home range) on movement patterns (described by home range crossing time, daily distance moved and directionality) and home range size of giant anteaters (*Myrmecophaga tridactyla*), as well as of the relationships between the descriptors of movement patterns to each other and with home range size. The standardized coefficients (β) represent the relative strength of significant effects.

We used mixed Structural Equations Modeling (mixed-SEM) [64] to investigate: (1) the effect of intrinsic traits (individuals’ sex and body mass) in movement patterns and home range size and (2) the effect of an environmental trait (proportion of forest cover inside the individuals’ home range) in movement patterns and home range size. Because the descriptors of movement patterns can be related to each other, and can modulate home range size, we controlled for these possible relationships in an integrative approach (see Fig 2). In this approach, the same variable could simultaneously act as response in an equation and as predictor in another one (Fig 2) [64]. Mixed-SEM allowed us to disentangle a complex net of interactions, estimating the indirect, direct, and total effects among variables [65]. Indirect effects were estimated by the product of the direct effects that compose them, and total effects were given by the sum of direct and indirect effects [66, 67].

Mixed-SEM was fitted using the PiecewiseSEM package [65, 68], available in the R environment [56]. We included random variables in the model to account for the hierarchical structure of our data (i.e., individuals’ intercepts were nested within the sites; see [65]). We also took into consideration the uncertainty associated with the estimated values of movement patterns and home range size. Accordingly, we used an autoregressive error structure to weigh the contribution of the values of the response variables for the inverse of its variance in the corresponding equations [69, 70]. We standardized the estimated coefficients to allow comparison between the different parameters [71]. Finally, we checked the global goodness-of-fit of our mixed-SEM by a Fischer’s C test, which measures the discrepancy between predicted and observed covariance matrices of our causal predictions [72].

## Results

### General results

We GPS-tracked 19 individuals–six individuals (three males and three females) in Santa Barbara Ecological Station (SP) in 2015, and 13 individuals (eight males and five females) in Baía das Pedras Ranch (MS) between 2013 and 2017. The individuals weighed between 21.6 kg and 38.7 kg (mean = 32.5 kg). Individuals’ body mass was similar for both study areas (t test; t = - 0.69; df = 12.53; p = 0.50). The GPS devices recorded location points at intervals ranging between 20 and 70 minutes. The monitoring time varied between individuals, ranging from 45 to 136 days in Santa Barbara Ecological Station (mean = 90 days) and from 69 to 509 days in Baía das Pedras Ranch (mean = 371.5 days). The total dataset consisted of 213,901 locations. We provided individual information on sex, body mass, sample regime and monitoring time in S1 Table.

The individuals’ empirical variogram showed the plotted semi-variance reaching an asymptote on a timescale that roughly corresponded to the home-range crossing time. Therefore, all the monitored giant anteaters showed constrained space use and were defined as range residents. For all individuals, the highest ranked movement model was the OUF–that takes into account autocorrelation in both location and velocity [26]. The estimates of home range crossing time, daily distance moved, directionality, and home range size varied between individuals (Tables 1 and S2). The mixed-SEM explained a substantial amount of the observed variation in home range crossing time (R² = 0.67), daily distance moved (R² = 0.72), directionality (R² = 0.88), and home range size (R² = 0.81).

**Table 1:** Estimates and confidence intervals of movement patterns (described by home range crossing time, daily distance moved and directionality) and home range size of giant anteaters (*Myrmecophaga tridactyla*).

	Minimum (95% CI)	Mean	Maximum (95% CI)
Home range crossing time (days)	0.26 (0.23–0.29)	2.15	10.58 (7.01–15.96)
Daily distance moved (km)	5.41 (3.74–7.08)	8.01	12.04 (11.90–12.19)
Directionality (min) ^a^	1.64 (0.94–2.87)	13.82	34.9 (31.76–38.37)
Home range (km²)	1.44 (1.09–1.84)	8.94	20.74 (15.26–27.06)

^a^ Directionality was measured as the timescale of the persistence in direction.

### Intrinsic effects on movement patterns and home range size

**Fig 3:**
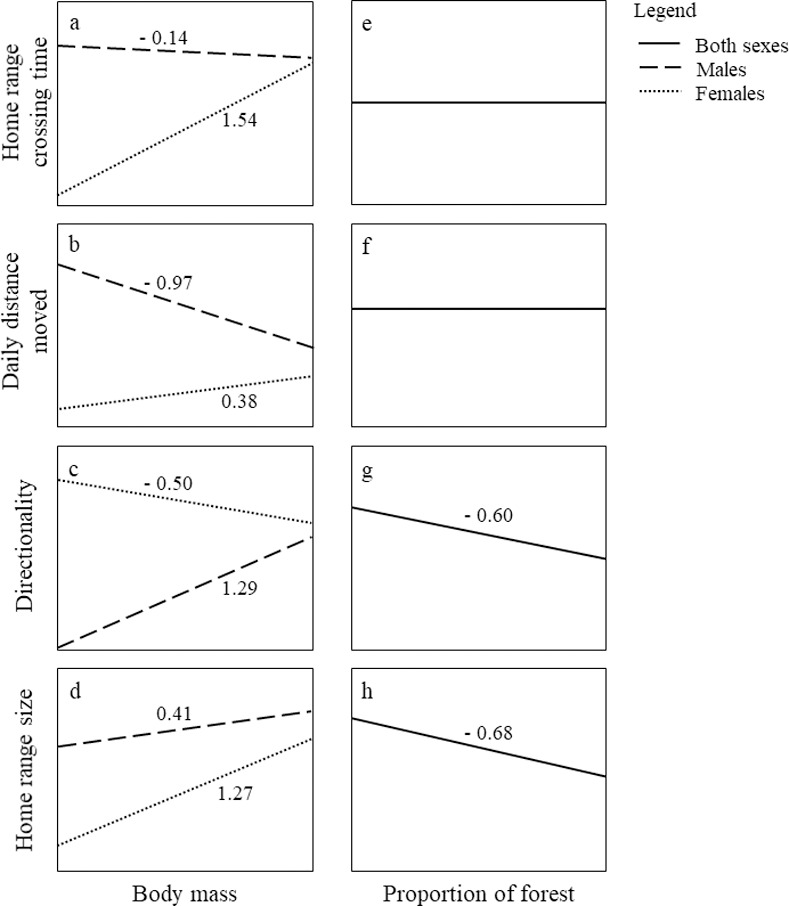
Graphical representation of the shape and direction of the effects of intrinsic and environmental traits on movement patterns and home range size of giant anteaters (*Myrmecophaga tridactyla*). Intrinsic traits are represented by sex and body mass. The environmental trait is represented by the proportion of forest cover within individuals’ home ranges. Movement patterns are described by home range crossing time, daily distance moved and directionality. Estimated coefficients are provided above tendency lines for each relationship.

Home range crossing time and daily distance moved had positive influence of sex, with males presenting higher values than females (Fig 2; β_1_ and β_2_, respectively; Fig 3A and 3B). The effect of body mass in home range crossing time and daily distance moved depended on the sex, and it was negative for males (Fig 2; β_3_ and β_5_, respectively; Fig 3A and 3B) and positive for females (Fig 2; β_4_ and β_6_, respectively; Fig 3A and 3B). Directionality was indirectly driven by sex through daily distance moved (Fig 2; β_2_* β_7_ = - 1.56; Fig 3C). The effect of body mass on directionality was also given indirectly via daily distance moved (Fig 2; β_5_* β_7_ for males, and β_6_* β_7_ for females), and it was equal to 1.29 for males and—0.50 for females (Fig 3C). The effect of both sex and body mass on home range size was mediated by home range crossing time, daily distance moved and directionality. The total effect of sex on home range size was given by β_1_* β_8_ + β_2_* β_7_* β_9_ = 0.45 (males > females; Figs 2 and 3D). The total effect of body mass on home range size was given by β_3_* β_8_ + β_5_* β_7_* β_9_ = 0.41 for males, and β_4_* β_8_ + β_6_* β_7_* β_9_ = 1.27 for females (Figs 2 and 3D).

### Environmental effect on movement patterns and home range size

The proportion of forest cover within the individuals’ home range ranged between 0.17 and 0.88 (mean = 0.42, S2 Table). The proportion of forest had no influence on home range crossing time or daily distance moved (Figs 2 and 3E and 3F), however, it negatively influenced directionality (Fig 2; β_10_ = - 0.60; Fig 3G). It means that individuals whose home range presented a higher proportion of forest cover showed less directionality–i.e., more tortuous movements–than individuals occupying areas with a low proportion of forests. The total effect of the proportion of forest cover on home range size resulted of the sum of its direct effect (Fig 2; β_11_) with indirect effects, mediated by directionality (Fig 2; β_10_* β_9_), totaling an effect of—0.68 (Figs 2 and 3H). In other words, individuals increased home range size with a decreasing proportion of forests inside it.

## Discussion

As far as we know, we provided here for the first-time movement model-based estimates of home range crossing time, daily distance moved and directionality for giant anteaters, allowing a better characterization of the species’ movement patterns. Once home range crossing time indicates the timescale of autocorrelation in position, our results show that, on average, a two-days interval between consecutive relocations is necessary to consider them spatially independent [63]. However, previous studies using GPS devices have adopted monitoring regimes much shorter than that and unconsidered the spatial autocorrelation of data [30, 42], probably leading to underestimating results. This is an important factor explaining why our estimates of daily distance moved, and home range size were, in average, bigger than those provided by recent studies [30, 42], once AKDEc incorporates and controls for the autocorrelation in both location and velocity [63]. Other possible explanations to this discrepancy can be related to the intrinsic characteristics of monitored individuals and the environmental characteristics associated with the site and period of monitoring.

The use of mixed-SEM allowed us to disentangle the effects of sex, body mass and proportion of forest on movement patterns and home range size, simultaneously estimating the direction and intensity of direct and indirect effects. The three descriptors of movement patterns (home range crossing time, daily distance moved and directionality) mediated the effects of sex and body mass on home range size, illustrating the importance of integrating these relationships in the same model [65]. We were able to clarify the sexual effects on movement patterns and home range size by considering body mass effects and the interactions between individuals’ sex and body mass. Even though the intraspecific effect of body mass on movement patterns and home range size is generally weak in mammals [12], we were still able to detect it with this integrative approach. To our best knowledge, this is the first record of allometric scaling in the movement patterns and home range size in giant anteaters. On the other hand, environmental traits, such as the proportion of forest cover, are common direct drivers of mammal’s home range size at the individual level [12], and our model provided additional details, showing the direct and indirect paths of this effect. Despite the great explanatory power of our model, we recognize that there must be other intrinsic and environmental factors influencing giant anteaters’ movement that we did not investigate here, and some of them may even seasonally change.

In general, male giant anteaters presented more intensive space use than females with similar body mass, showing longer home range crossing-time, longer daily distance moved and smaller directionality. Besides, males also exhibited larger home ranges than females. We expected males moving longer distances and occupying larger areas than females. However, it was surprising that they also took more time to cross their areas and were less directional at doing it. The higher intensity of space use and larger home range in males than females are, probably, strategies to increase the chances to find receptive females on landscape [44]. This is because the home range of a male giant anteater usually overlaps with the home range of several females [73]. Hence, males could increase their chances of finding receptive females exploiting their home ranges and increasing their home ranges size to include more females inside it. In line with these results, male giant anteaters were recorded presenting longer activity time and larger home range than females [42]. Therefore, we reinforced the idea that giant anteaters present a polygynous social mating system, with a male mating with more than one female [43, 44].

Female giant anteaters behaved as expected, increasing the intensity of space use with increasing body mass. This is probably related to an increase in the search for food resources [26], once larger animals have higher energetic requirements [45]. For species that have their food resources unpredictably spread on the landscape, such as the invertebrate nests that giant anteaters prey upon, the intensity of utilization of food resources depends on the intensity of use of space that provides physical access to those resources [74, 75]. Besides, female giant anteaters also increased home range size with increasing body mass, showing a second strategy to increase the access to food resources: increasing the size of the space used to find those resources. A positive allometric scaling of both intensity of space use and home range size has been found in some mammals [76, 77], including other xenarthrans with myrmecophagous diets such as giant armadillos (*Priodontes maximus*) [78] and southern three-banded armadillos (*Tolypeutes matacus*) [79]. This relationship indicates that the search for energetic resources is one of the main factors driving female giant anteaters’ movement across body mass.

On the other hand, males did not display the same pattern. With increasing body mass, males reduced intensity of space use and increased home range size. This reveals a change of males’ movement strategy guided by body mass: while small males used their small areas intensively, large males ranged over large areas with comparatively lower intensity of use. Considering a limited quantity of metabolic energy available for movement [80], animals moving close to their limit capacity should experiment a trade-off between the intensity of use and the area size, and this can be the case of male giant anteaters. Both strategies can increase the access to both food resources spread on the landscape and receptive females [81]. Meanwhile, small males could minimize the chances of agonistic interactions with other males if they use smaller areas than the big ones [82, 83]. Further studies, such as behavioral assessments, will help us to confirm these hypotheses and better understand the species’ reproductive biology.

As we expected, male and female giant anteaters reduced the directionality as the proportion of forest patches inside their home ranges increased, probably due to the physical obstacles that forests impose to displacement [84]. Similarly, small mammals have presented shorter step lengths and higher tortuosity within forest areas [85], and African wild dogs have shown that the movement permeability of the vegetation decreases with its increasing density [86]. However, it is worth noting that, contrary to our expectations, a greater proportion of forest inside the home ranges did not influence the home range crossing time or daily distance moved.

Decreasing the proportion of forest inside giant anteaters’ home range led males and females to an increase in the home range size. This is probably because giant anteaters present a low capacity for physiological thermoregulation [37], and less forest implies less access to thermal shelters and, consequently, a reduced habitat thermal quality [34–36]. As a result, animals would increase the home range size as a strategy to maximize the chances of accessing this thermal resource. Supporting this idea, the increase of home range size with decreasing habitat quality has been widely documented for terrestrial vertebrates [87–89]. Furthermore, the importance of forests as thermal shelters has also been shown for other mammals [90, 91], and it should increase with the predicted increasing frequency of extreme weather events [92]. In the Brazilian territory, where this study was conducted, massive agricultural expansion has caused extensive habitat degradation and dramatically decreased forest patches on savannah areas in number and size [93, 94]. In this current deforestation scenario, our results bring an important implication for giant anteaters’ management: the minimal area needed to preserve a given giant anteaters’ population should increase as the proportion of forests inside it decreases.

In this study, we brought reliable measures of giant anteaters’ movement patterns and home-range size, showing that their movements are influenced by sex, body mass and proportion of forest cover; and revealed two important strategies used by giant anteaters to maximize the access to resources: they modulate movement patters, increasing space use intensity, and/or increasing home range size. This information contributes to the understanding of giant anteaters’ spatial ecology and can help define the spatial scale of effective management efforts for their conservation [95], especially as the anthropogenic impacts on landscapes increase. We highlight the need to consider the sexual differences on movement strategies and the role of forests as an important thermal resource driving giant anteaters’ spatial requirements (also see [96]). In accordance with [36], we strongly suggest that management efforts should focus on maintaining the giant anteaters’ access to forest patches inside their home ranges to provide environmental conditions for behavioral thermoregulation. Both intrinsic and environmental traits driving animal movement should be integrated when establishing conservation strategies for populations and species.

## Supporting information

S1 Table Intrinsic characteristics and monitoring information of tracked giant anteaters. (DOC)

S2 Table Individual estimates of movement patterns, home range size and proportion of forests inside home range of tracked giant anteaters. (DOC)
