# Supplementary material for: Sexual, allometric and forest cover effects on giant anteaters’ movement ecology
Source: PLoS One. 2021 Aug 18;16(8):e0253345. doi: 10.1371/journal.pone.0253345 (PMC8372905; doi:10.1371/journal.pone.0253345)
Supplement: S1 Table — (DOC) [file pone.0253345.s001.doc]

**S1 Table. Intrinsic characteristics and monitoring information of tracked giant anteaters.**

| Id | Site | Sex | Body Mass (kg) | Sampling Regime (minutes) | Monitoring time (days) | Number of points |
| --- | --- | --- | --- | --- | --- | --- |
| 1 | MS | F | 30 | 20 | 84.03 | 4529 |
| 2 | MS | F | 39 | 20 | 386.04 | 27162 |
| 3 | MS | F | 26.3 | 30 | 339.72 | 11857 |
| 4 | MS | F | 30 | 20 | 69.06 | 788 |
| 5 | MS | F | 28.1 | 20 | 159.94 | 8717 |
| 6 | MS | M | 30 | 20 | 144.73 | 10273 |
| 7 | MS | M | 35 | 20 | 363.16 | 25866 |
| 8 | MS | F | 33.3 | 20 | 122.45 | 8730 |
| 9 | MS | M | 36 | 20 | 509.74 | 32908 |
| 10 | MS | M | 37.2 | 30 | 387.77 | 16733 |
| 11 | MS | F | 35 | 30 | 279.75 | 11604 |
| 12 | MS | M | 35 | 20 | 378 | 27139 |
| 13 | MS | M | 25 | 20 | 365 | 17178 |
| 14 | SP | F | 34.8 | 40 | 44.6 | 839 |
| 15 | SP | M | 35.2 | 60 | 136.08 | 2467 |
| 16 | SP | M | 36.6 | 40 | 90 | 1608 |
| 17 | SP | F | 33 | 60 | 107.63 | 2019 |
| 18 | SP | M | 36.2 | 70 | 106.38 | 2091 |
| 19 | SP | F | 21.6 | 60 | 80.27 | 1393 |
