# Supplementary material for: Sexual, allometric and forest cover effects on giant anteaters’ movement ecology
Source: PLoS One. 2021 Aug 18;16(8):e0253345. doi: 10.1371/journal.pone.0253345 (PMC8372905; doi:10.1371/journal.pone.0253345)
Supplement: S2 Table — (DOC) [file pone.0253345.s002.doc]

**S2 Table.** **Individual estimates of movement patterns, home range size and proportion of forests inside home range of tracked giant anteaters.**

| Id | Home range crossing time (days) (95% CI) | Daily distance moved (km) (95% CI) | Directionality (min) (95% CI) | Home range (km²) (95% CI) | Proportion of forest |
| --- | --- | --- | --- | --- | --- |
| 1 | 1.78 (1.25 - 2.53) | 6.50 (4.98 - 11.15) | 1.64 (0.94 - 2.87) | 9.10 (6.62 - 11.96) | 0.4173 |
| 2 | 10.58 (7.01 - 15.96) | 7.80 (7.74 - 7.86) | 8.55 (8.32 - 8.87) | 22.60 (15.64 - 30.83) | 0.2942 |
| 3 | 1.29 (1.12 - 1.47) | 7.20 (7.12 - 7.29) | 14.40 (13.85 - 14.97) | 4.78 (4.21 - 5.39) | 0.4534 |
| 4 | 0.46 (0.33 - 0.62) | 6.80 (5.20 - 12.82) | 2.77 (0.56 - 13.64) | 4.94 (3.85 - 6.17) | 0.4527 |
| 5 | 1.66 (1.32 - 2.09) | 10.77 (10.61 - 10.92) | 8.32 (7.62 - 8.74) | 8.90 (7.19 - 10.79) | 0.3065 |
| 6 | 0.95 (0.79 - 1.13) | 7.65 (7.57 - 7.73) | 11.06 (10.65 - 11.50) | 3.80 (3.21 - 4.43) | 0.3359 |
| 7 | 3.54 (2.83 - 4.43) | 9.77 (9.70 - 9.85) | 8.71 (8.47 - 8.95) | 13.20 (10.70 - 15.97) | 0.2388 |
| 8 | 0.92 (0.75 - 1.11) | 7.09 (7.00 - 7.17) | 9.68 (9.25 - 10.12) | 2.34 (1.95 - 2.76) | 0.3376 |
| 9 | 2.86 (2.42 - 3.37) | 9.50 (9.45 - 9.60) | 7.80 (7.60 - 8.01) | 9.74 (8.32 - 11.28) | 0.3041 |
| 10 | 2.11 (1.80 - 2.48) | 7.90 (7.83 - 7.96) | 17.33 (16.83 - 17.84) | 11.86 (10.18 - 13.66) | 0.2286 |
| 11 | 2.19 (1.80 - 2.67) | 7.86 (7.78 - 7.93) | 19.06 (18.42 - 19.72) | 12.00 (9.90 - 14.21) | 0.1698 |
| 12 | 3.03 (2.67 - 4.01) | 8.06 (8.01 - 8.11) | 12.13 (11.90 - 12.40) | 12.68 (10.40 - 15.24) | 0.4338 |
| 13 | 3.89 (3.08 - 4.89) | 12.04 (11.90 - 12.19) | 7.31 (7.01 - 7.62) | 18.24 (14.70 - 22.16) | 0.1926 |
| 14 | 2.41 (1.21 - 4.78) | 7.59 (6.79 - 8.39) | 15.28 (10.96 - 21.32) | 10.39 (5.98 - 16.01) | 0.5242 |
| 15 | 1.30 (1.02 - 1.64) | 8.41 (8.01 - 8.81) | 18.25 (15.74 - 21.16) | 8.31 (6.75 - 10.04) | 0.7095 |
| 16 | 1.19 (0.89 - 1.58) | 5.87 (5.69 - 6.05) | 32.10 (28.63 - 35.99) | 6.83 (5.31 - 8.55) | 0.7257 |
| 17 | 0.66 (0.54 - 0.80) | 5.44 (5.26 - 5.61) | 27.17 (24.15 - 30.58) | 2.75 (2.32 - 3.21) | 0.6086 |
| 18 | 2.05 (1.45 - 2.89) | 8.11 (7.91 - 8.31) | 34.90 (31.76 - 38.37) | 20.74 (15.26 - 27.06) | 0.4282 |
| 19 | 1.29 (0.94 - 1.77) | 5.41 (3.74 - 7.08) | 6.07 (1.89 - 19.48) | 1.44 (1.09 - 1.84) | 0.8795 |
